# Autologous minced muscle grafts improve endogenous fracture healing and muscle strength after musculoskeletal trauma

**DOI:** 10.14814/phy2.13362

**Published:** 2017-07-26

**Authors:** Brady J. Hurtgen, Catherine L. Ward, Chrissy M. Leopold Wager, Koyal Garg, Stephen M. Goldman, Beth E. P. Henderson, Todd O. McKinley, Sarah M. Greising, Joseph C. Wenke, Benjamin T. Corona

**Affiliations:** ^1^ Extremity Trauma and Regenerative Medicine Task Area US Army Institute of Surgical Research Fort Sam Houston Texas; ^2^ Department of Orthopaedic Surgery Indiana University School of Medicine Indianapolis Indiana

**Keywords:** Inflammation, insulin‐like growth factor‐1, orthopedic trauma, regenerative medicine, skeletal muscle injury, volumetric muscle loss

## Abstract

The deleterious impact of concomitant muscle injury on fracture healing and limb function is commonly considered part of the natural sequela of orthopedic trauma. Recent reports suggest that heightened inflammation in the surrounding traumatized musculature is a primary determinant of fracture healing. Relatedly, there are emerging potential therapeutic approaches for severe muscle trauma (e.g., volumetric muscle loss [VML] injury), such as autologous minced muscle grafts (1 mm^3^ pieces of muscle; GRAFT), that can partially prevent chronic functional deficits and appear to have an immunomodulatory effect within VML injured muscle. The primary goal of this study was to determine if repair of VML injury with GRAFT rescues impaired fracture healing and improves the strength of the traumatized muscle in a male Lewis rat model of tibia open fracture. The most salient findings of the study were: (1) tibialis anterior (TA) muscle repair with GRAFT improved endogenous healing of fractured tibia and improved the functional outcome of muscle regeneration; (2) GRAFT repair attenuated the monocyte/macrophage (CD45^+^ CDllb^+^) and T lymphocyte (CD3^+^) response to VML injury; (3) TA muscle protein concentrations of MCP1, IL‐10, and IGF‐1 were augmented in a proregenerative manner by GRAFT repair; (4) VML injury concomitant with osteotomy induced a heightened systemic presence of alarmins (e.g., soluble RAGE) and leukocytes (e.g., monocytes), and depressed IGF‐1 concentration, which GRAFT repair ameliorated. Collectively, these data indicate that repair of VML injury with a regenerative therapy can modulate the inflammatory and regenerative phenotype of the treated muscle and in association improve musculoskeletal healing.

## Introduction

Volumetric muscle loss (VML) injuries occur frequently in civilian and military orthopedic trauma. This destructive form of injury results in a permanent loss of muscle tissue and strength (Corona et al. 2016), heightened and prolonged muscle inflammation (Hurtgen et al. 2016; Sadtler et al. 2016), extensive compartmental fibrosis (Garg et al. 2014a), loss of range of motion (Garg et al. 2015), disrupted intrinsic muscle properties (Corona et al. 2013a; Garg et al. 2015), and gross limb dysfunction (Garg et al. 2015). Together, these manifestations of VML injury contribute to chronic disability (Corona et al. 2015; Rivera and Corona 2016). Perhaps less appreciated, VML injury that occurs simultaneously with adjacent diaphyseal fracture (i.e., open fracture) impairs bone regeneration (Karladani et al. 2001; Papakostidis et al. 2011). In the extreme, complete ablation of the tibialis anterior (TA) muscle in rats delayed endogenous healing of a simple tibia osteotomy by a mechanism unrelated to fracture site revascularization (Utvag et al. 2002, 2003). Interestingly, even a relatively small partial resection (i.e., 10–20% frank removal) of local muscle tissue impaired endogenous healing of a rat tibia osteotomy or rhBMP‐2 mediated bone regeneration within segmental defects in the femur and tibia (Willett et al. 2013; Hurtgen et al. 2016; Pollot et al. 2016), suggesting that perturbations of the local wound environment caused by VML injury impairs musculoskeletal regeneration.

The current surgical standard of care for long bone fracture with concomitant VML injury generally includes debridement, fracture fixation, and skin closure. Direct regenerative therapies are not applied to the traumatized muscle, although in severe cases muscle or fasciocutaneous flaps may be used to restore soft tissue coverage of the fractured bone. The intent of the flapped tissue is to support fracture healing by (1) mitigating secondary contamination and (2) re‐establishing a vascularized tissue bed that may nourish the fracture site and remove metabolic waste materials (Richards and Schemitsch 1989; Khodaparast et al. 2003), but not to restore muscle function. Muscle flaps in particular provide myokines and myogenic stem cells that promote fracture healing by re‐establishing endogenous muscle‐bone interactions (Chan et al. 2012). Lastly, muscle flaps may also ameliorate fracture healing by damping the local immune response (Brown et al. 2000), and therefore alleviate inhibitory effects of heightened inflammation on bone regeneration at the fracture site.

Ideally, a regenerative therapy would be available to the orthopedic surgeon to acutely repair VML injuries concomitant to fracture. The intention of the therapy would be to support fracture healing, like a muscle flap, and to improve muscle function. Currently developing therapies for VML injury can be described by the degree to which critical components of skeletal muscle (e.g., basal lamina and satellite cells), or their putative surrogates, are implanted. For example, acellular biological extracellular matrix devices, which principally provide a provisional matrix for host cell migration and tissue formation, embody a single component approach (for review see Wolf et al. 2015). Alternatively, autologous minced muscle grafts (~1 mm^3^ pieces of muscle tissue; GRAFT), which are comprised of all resident regenerative components in mammalian skeletal muscle, embodies a multiplex therapy (Studitsky 1964; Carlson 1968, 1972; Carlson and Gutmann 1972; Corona et al. 2013a). Despite varying degrees of success in regenerating functional muscle tissue, both acellular biological scaffolds and autologous grafts can improve muscle function, promote the formation of a vascularized tissue bed infiltrated with Sca1^+^ or CD146^+^ putative stem cells, and appear to protect the remaining musculature from secondary or recurrent damage and prolonged inflammation (Corona et al. 2013a, 2013b; Garg et al. 2014b; Aurora et al. 2015; Ward et al. 2015, 2016; Corona and Greising 2016; Kasukonis et al. 2016; Sadtler et al. 2016). Given these properties, it is plausible that direct repair of a VML injury with a regenerative therapy will stabilize the local wound environment and ameliorate the deleterious effect of concomitant muscle trauma on fracture healing.

Recently, an acellular biological scaffold was investigated as a dual‐purpose VML injury therapy in a rat open fracture model (i.e., 3 mm tibia segmental defect treated with rhBMP‐2 with TA muscle VML injury) (Pollot et al. 2016). Contrary to some previous observations (Corona et al. 2013b; Aurora et al. 2015), the scaffold did not improve muscle function impaired by VML. Moreover, the scaffold exacerbated the impairment of rhBMP‐2 mediated bone regeneration observed with non‐repaired VML injury (Pollot et al. 2016). While the direct cause of these negative results was not completely determined, distinct cortical bone resorption suggested elevated osteoclast activity secondary to exacerbated inflammation. Given these results, the purpose of this study was to test the hypothesis that a multiplex regenerative approach, namely autologous minced muscle grafts, can serve as a dual‐purpose therapy for VML injury and fracture healing. Upon observing that autologous minced graft repair of VML injury improved musculoskeletal healing outcomes, local and systemic markers of inflammation and regeneration (i.e., Insulin‐like growth factor‐1; IGF‐1) were subsequently investigated to determine the capacity of a direct muscle therapy to modulate the local wound environment towards a proregenerative phenotype. This work was performed in an established muscle‐bone composite injury model in adult male Lewis rats (Hurtgen et al. 2016).

## Methods

### Animals

All animal procedures were approved by the Institutional Animal Care and Use Committee and were conducted in compliance with the Animal Welfare Act and in accordance with the principles of the Guide for the Care and Use of Laboratory Animals. Inbred male Lewis rats (350–400 g; ~4 months of age) were purchased from Envigo (Harlan) Laboratories (Huntingdon, United Kingdom) and housed in a specific pathogen‐free animal facility. All rats received a presurgical (~30 min prior) administration of buprenorphine‐SR (1.2 mg/kg; s.c.) for pain management and a postsurgical x‐ray was taken to ensure proper fracture fixation. All rats were observed twice daily for the first 3 days postinjury and at least weekly thereafter for signs of distress and lack of mobility, no adverse complications were observed.

### Experimental design

Male Lewis rats underwent surgical creation of an endogenously healing tibia osteotomy (OST) with or without concomitant ipsilateral TA muscle VML injury (OST + VML). Briefly, the OST was performed approximately 5 mm proximal to the tibia‐fibula junction and stabilized with a 1.25 mm Kirschner pin inserted in the medullary cavity from the tibial plateau after reaming (Hurtgen et al. 2016). VML injury was surgically created in the middle third (10 mm distal from the tibial tuberosity) of the adjacent TA muscle using a punch biopsy (6 mm) and was then marked on each border with 6‐0 prolene suture, as we have reported previously (Corona et al. 2013a; Garg et al. 2015). In a subset of rats, the VML injured muscle was immediately repaired with autologous minced muscle grafts (1 mm^3^ pieces of skeletal muscle; GRAFT) that were derived from the portion of the muscle excised (Carlson 1968; Corona et al. 2013a). Injured rats were therefore allotted to the following experimental groups: OST (no VML), OST + VML (VML, no repair), and OST + VML + GRAFT. Rats were followed out to 28 days postinjury, at which time musculoskeletal physiological and mechanical assessments were performed. To investigate associations between healing outcomes and the early immune response TA muscle and serum were harvested 3 and 14 days postinjury to perform flow cytometric, histological, and protein expression analyses in a subset of animals. A portion of the control group data presented were reported previously (Hurtgen et al. 2016); all experimental data were collected during the same time period.

### Micro‐computed tomography

Microcomputed tomography (*μ*CT) scans (Viva‐CT40; Scanco Medical, Brüttisellen, Switzerland) were performed on rat tibias collected at 28 days postinjury. Tibiae were arranged longitudinally to the x‐ray source in porous foam. Osteotomy regions were scanned at medium resolution with a 21 *μ*m voxel size at a voltage of 55 kVp and a current of 145 *μ*A. A volume of interest of 400 slices was analyzed (~8.4 mm) with a centralized osteotomy site. Bone and soft tissue were segmented by applying a threshold corresponding to 455 mg hydroxyapatite/cm^3^ and a low‐pass Gaussian filter (sigma = 1.2, support = 1.0) was applied to suppress noise. Contours were first drawn around the entire specimen to obtain a total volume and then drawn around the cortical bone to obtain bone (original) volume. Callus volume was calculated by subtracting bone (original) volume from total volume, in which the mineralized content is reported.

### Bone mechanical testing

Three‐point bending was performed on a set of rat tibiae at 28 days postinjury, using methods previously described (Hurtgen et al. 2016). Load‐deflection curves were used to obtain maximum load and stiffness.

### Muscle functional assessment

In vivo functional testing of TA muscles was performed at 28 days postinjury using methodology previously described (Corona et al. 2013a; Garg et al. 2014b, 2015). Briefly, TA muscle in vivo physiological properties were measured in anesthetized rats (isoflurane 1.5–2.0%) using a dual‐mode muscle lever system (Aurora Scientific, Inc.; Aurora, Canada: Mod. 305b). Subcutaneous needle electrodes were inserted in the lateroposterior aspect of the lower limb on each side of the common peroneal nerve. Optimal voltage (2–5 V) was set with a series of tetanic contractions (5–10 contractions; 150 Hz, 0.1 ms pulse width, 400 ms train). Then, a skin incision was made at the anterolateral aspect of the ankle and the distal tendons of the extensor digitorum longus and extensor hallicus longus (EHL) muscles was isolated and severed above the retinaculum. Previous pilot testing indicated that the contribution of the EDL muscle to net torque is negligible under these conditions (Corona et al. 2013a). TA muscle maximal isometric tetanic torque was measured as a function of stimulation frequency (10–200 Hz) with the ankle at a right angle.

### TA muscle histology

TA muscle tissue isolation and freezing was performed as described previously (Corona et al. 2013a; Garg et al. 2014b). Tissue sections (10 *μ*m thickness) were stained with hematoxylin and eosin (H&E). Composite images of the complete TA muscle were acquired using Axio Scan.Z1 microscope and ZEN imaging software (Carl Zeiss Microscopy; Jena, Germany). Sections were also examined by brightfield microscopy (Olympus IX71; Olympus; Tokyo, Japan) and images of muscle tissue were acquired and analyzed using cellSens Standard software (Ver 1.4.1, Olympus).

### Muscle and serum protein measurements

To analyze protein levels of various analytes, enzyme‐linked immunosorbent assays (ELISA) and Bio‐Plex assays were conducted using sera or protein extracted from TA muscle tissue. To isolate sera, whole blood was collected by cardiac puncture and allowed to clot on ice for 30 min before centrifugation. Total protein was extracted from approximately 50 mg of TA muscle tissue (midbelly) using a Bio‐Plex Cell Lysis Kit (Bio‐Rad; Hercules, CA). A protease inhibitor cocktail (Sigma‐Aldrich; St. Louis, MO) was added to sera and protein extracts before storage at −80°C. Immediately prior to analysis, protein was quantified using the bicinchoninic acid (BCA) assay (Pierce Biotechnology; Waltham, MA). High mobility group box 1 (HMGB1) and soluble receptor for advanced glycation end products (sRAGE) were both detected in serum by ELISA (Chondrex, Inc.; Redmond, WA and Abcam; Cambridge, UK, respectively). IGF‐1 levels were detected in sera and protein extracts using the mouse/rat IGF‐1 immunoassay kit as described by the manufacturer's protocol (R&D Systems; Minneapolis, MN). Tissue levels of select chemokines and cytokines, monocyte chemoattractrant protein‐1 (MCP‐1), tumor necrosis factor‐*α* (TNF‐*α*), interleukin‐6 (IL‐6), and inerleukin‐10 (IL‐10) were detected using the BioPlex Pro rat cytokine multi‐plex immunoassay (Bio‐Rad) according to manufacturer's protocol.

### Cellular isolation from muscle tissue

Cells were isolated from the middle third of the TA muscle that encompassed the defect by enzymatic digestion as previously described (Hurtgen et al. 2016). Briefly, the defect was surgically isolated and the mass was determined. Tissue was incubated with collagenase type II and dispase for 90 min at 37°C. Cells were further released by gentle mechanical disruption and filtered through a 70 *μ*m cell strainer. Erythrocytes were lysed with ammonium‐chloride‐potassium lysing buffer and cells were filtered through a 40 *μ*m cell strainer, washed, and resuspended in PBS containing 0.5% FBS and 0.1% sodium azide. Viable cells were quantified using trypan blue exclusion on a hemocytometer.

### Quantification of cellular infiltrates in muscle defect by flow cytometry

Muscle‐derived cells were labeled with fluorochrome‐conjugated antibodies and analyzed by flow cytometry as previously described (Hurtgen et al. 2016). Cells were incubated with anti‐CD32 antibody to block Fc receptors and labeled with either cocktail monoclonal antibodies to identify macrophages or T lymphocytes. The macrophage (MØ) cell cocktail included anti‐CD45 (clone OX‐1), anti‐CD11b (WT.5), anti‐CD86 (24F), and anti‐CD163 (ED2). The T lymphocyte cell cocktail consisted of anti‐CD45, anti‐CD3 (IF4), anti‐CD4 (OX‐35), and anti‐CD8*α* (OX‐8) (Table 1). Labeled cells were fixed with 1% paraformaldehyde and enumerated by fluorescence‐activated cells sorting using a MACSQuant flow cytometer (Miltenyi Biotec; Bergisch Gladbach, Germany). Data were analyzed using MACSQuantify software (Miltenyi Biotec). Gating strategies (see Hurtgen et al. 2016) to identify cellular populations included the following: CD45^+^ hematopoietic cells, CD45^+^CD11b^+^ MØ, CD45^+^CD11b^+^CD86^+^ M1‐like MØ, CD45^+^CD11b^+^CD163^+^ M2‐like MØ, CD45^+^CD3^+^CD4^+^, and CD45^+^CD3^+^CD8*α* ^+^ T lymphocytes. Cell numbers of each gated population were determined by multiplying the percentage of cells by the total number of viable cells recovered from the respective defect. All cell numbers were normalized per gram of excised muscle tissue and these values were used for statistical comparisons among groups.

**Table 1:** Flow cyotometry antibodies

Antigen	Antibody clone	Vendor	Catalog number	Concentration
CD3	IF4	BD Biosciences	557030	0.001 mg/mL
CD4	OX‐35	BD Biosciences	554838	0.001 mg/mL
CD8a	OX‐8	BD Biosciences	561965	0.001 mg/mL
CD11b	WT.5	BD Biosciences	554982	0.001 mg/mL
CD45	OX‐1	BD Biosciences	561586	0.001 mg/mL
CD68	ED1	Novus Biologicals	NB600‐985V	0.001 mg/mL
CD86	24F	BD Biosciences	551396	0.001 mg/mL
CD163	ED2	Bio‐Rad	MCA342A647	0.001 mg/mL
CD32	C34‐485	BD Biosciences	550271	0.0025 mg/mL

### Circulating blood cell analysis

Whole blood was collected by cardiac puncture and placed in tubes coated with lithium heparin. White blood cell counts were determined using a Coulter Ac‐T diff2™ hematology analyzer (Beckman Coulter, Inc.; Brea, CA).

### Statistics

Dependent variables were analyzed using one‐ or two‐way ANOVA or Student's *t*‐test. In the event of a significant ANOVA, Fisher's post hoc testing was performed. Statistical significance was achieved at alpha of 0.05. Statistical testing was performed using Prism 6 for Mac OSX (Graphpad Inc; La Jolla, CA). Only statistically significant measurements are described in the results, with all variables measured referenced in the corresponding figures or Table 2. Percent increases or decreases between specified groups are used in the text to specify direction and magnitude of responses. The units of each variable measured are listed in Table 2. Sample sizes for *μ*CT, bone mechanical testing, and in vivo TA muscle functional testing were 6–8 per group. Histology, flow cytometry, cytokine analyses, and blood leukocyte analyses were performed with sample sizes of 4–6 per group and time point.

**Table 2:** Bone, muscle, and systemic indices of healing, inflammation, and anabolism

Dependent measure	Method	Group mean ± SEM			% Change from OST	
		OST	OST + VML	OST + VML + GRAFT	OST + VML	OST + VML + GRAFT
Bone regeneration (28d TPI)						
Mineralized bone (mm^3^)	*μ*CT	15.8 ± 1.5	9.1 ± 1.8*	13.9 ± 1.2†	−42.6	−12.1
Max load (N)	Three point bending	30.8 ± 5.7	13.9 ± 3.6*	32.5 ± 10.8†	−55.0	5.5
Stiffness (N/mm)	Three point bending	101.5 ± 12.8	27.8 ± 10.9*	93.2 ± 23.5†	−72.6	−8.1
Muscle regeneration (28d TPI)						
TA muscle Torque @ 150 hz (N/mm)	In vivo function	2.4 ± 0.1	1.5 ± 0.1*	1.9 ± 0.01*, †	−37.4	−23.5
Muscle inflammation (3 & 14d TPI)						
3d TPI						
CD45^+^ cells (×10^6^)	Flow cytometry	5.9 ± 2.2	23.3 ± 2.2*	14.3 ± 0.6*, †	292.7	141.2
CD11b^+^ cells (×10^6^)	Flow cytometry	5.6 ± 2.1	21.4 ± 2.0*	13.4 ± 0.6*, †	286.1	140.5
CD86^+^ cells (×10^6^)	Flow cytometry	2.0 ± 0.7	7.2 ± 1.2*	3.8 ± 0.1*, †	263.3	92.0
CD163^+^ cells (×10^6^)	Flow cytometry	2.1 ± 0.7	8.5 ± 1.2*	4.7 ± 0.4*, †	312.1	130.1
CD3^+^ cells (×10^5^)	Flow cytometry	1.0 ± 0.2	4.0 ± 0.1*	2.8 ± 0.6*, †	289.2	171.6
CD4^+^ cells (×10^5^)	Flow Cytometry	0.7 ± 0.2	2.7 ± 0.2*	1.7 ± 0.3*, †	283.0	144.3
CD8^+^ cells (×10^5^)	Flow cytometry	0.2 ± 0.1	0.7 ± 0.1*	0.6 ± 0.2	295.3	221.3
MCP‐1 (pg/*μ*g)	ELISA	9.2 ± 2.0	55.3 ± 5.3*	28.8 ± 4.3*, †	503.2	214.4
TNF‐*α* (pg/*μ*g)	ELISA	52.0 ± 7.0	59.4 ± 6.3	49.7 ± 12.2	14.2	−4.4
IL‐6 (pg/*μ*g)	ELISA	53.6 ± 2.5	59.9 ± 3.0	47.2 ± 3.5†	11.7	−11.9
IL‐10 (pg/*μ*g)	ELISA	47.1 ± 6.5	19.7 ± 4.2*	47.7 ± 8.8†	−58.1	1.3
14d TPI						
CD45^+^ cells (×10^6^)	Flow cytometry	0.3 ± 0.1	1.6 ± 0.2	2.3 ± 0.3	537.9	801.6
CD11b^+^ cells (×10^6^)	Flow cytometry	0.2 ± 0.1	1.4 ± 0.2	1.8 ± 0.2	581.5	799.5
CD86^+^ cells (×10^6^)	Flow cytometry	0.1 ± 0.1	0.6 ± 0.1	0.7 ± 0.1	472.4	528.6
CD163^+^ cells (×10^6^)	Flow cytometry	0.1 ± 0.1	1.1 ± 0.2	1.5 ± 0.2	667.1	957.1
CD3^+^ cells (×10^5^)	Flow cytometry	0.2 ± 0.1	1.4 ± 0.1*	2.4 ± 0.4*, †	479.3	896.6
CD4^+^ cells (×10^5^)	Flow cytometry	0.1 ± 0.1	0.7 ± 0.1*	1.2 ± 0.3*, †	535.2	1078.1
CD8^+^ cells (×10^5^)	Flow cytometry	0.1 ± 0.1	0.1 ± 0.1	0.4 ± 0.1*	114.8	2533.3
Systemic Inflammation (3d TPI)						
sRAGE (pg/*μ*L)	ELISA	117.4 ± 2.6	153.8 ± 14.8*	109.6 ± 4.4†	31.0	−6.6
HMGB1 (pg/*μ*L)	ELISA	74.1 ± 11.5	124.1 ± 26.2	70.5 ± 9.5	67.4	−4.9
Monocytes (cells/*μ*L × 10^3^)	CBC	0.7 ± 0.1	1.3 ± 0.1*	1.0 ± 0.1	85.7	42.9
Granulocytes (cells/*μ*L × 10^3^)	CBC	0.4 ± 0.7	0.6 ± 0.2	0.4 ± 0.03	50.0	0.0
Lymphotyces (cells/*μ*L × 10^3^)	CBC	4.1 ± 0.6	5.8 ± 0.3*	5.5 ± 0.5*	41.5	34.1
Anabolic response (3d TPI)						
IGF‐1 muscle (pg/*μ*g)	ELISA	31.7 ± 7.6	57.1 ± 9.0*	80.9 ± 8.3*, †	80.1	155.3
IGF‐1 serum (ng/mL)	ELISA	516.5 ± 81.1	223.4 ± 30.16*	488.1 ± 96.3†	−56.7	−5.5

TPI, time postinjury; VML, volumetric muscle loss; OST, osteotomy.

* ≠ OST, ^†^ ≠ OST + VML: *P* < 0.05.

## Results

### Fracture healing

**Figure 1:**
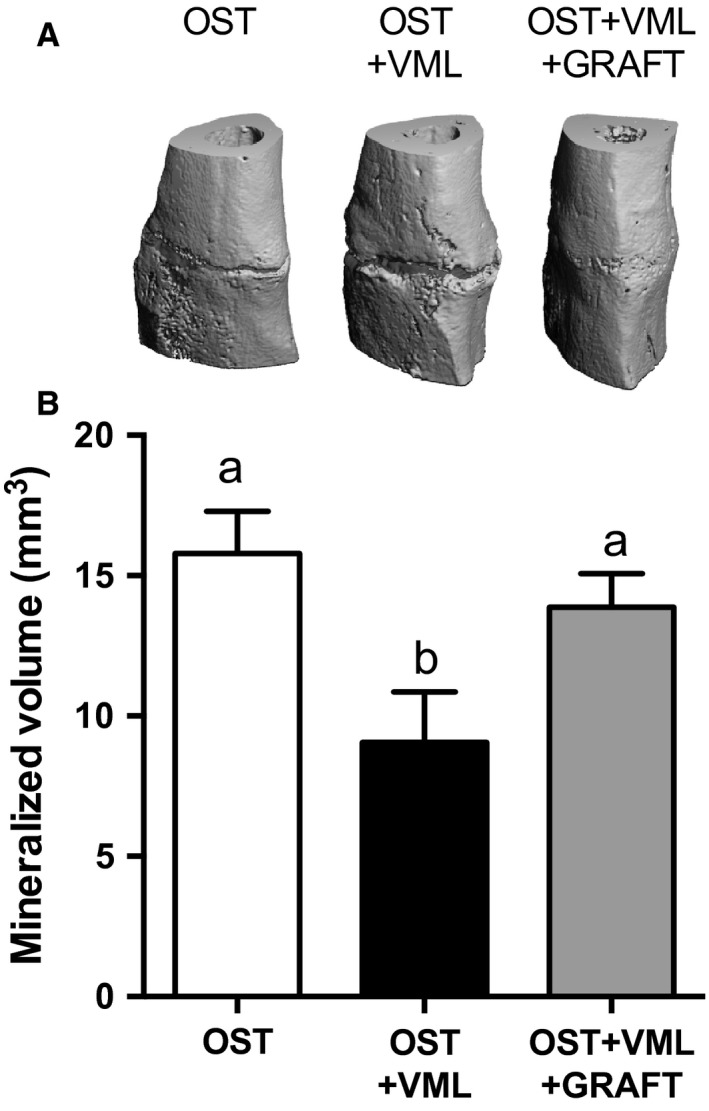
Autologous minced muscle grafts restores aggregation of mineralized content at the site of fracture following severe musculoskeletal trauma. (A) Digitalized fracture site renderings acquired by *μ* CT 28 days postinjury from tibia osteotomy (OST) and osteotomy + tibialis anterior (TA) muscle volumetric muscle loss (VML) injury with no repair (OST + VML) or minced graft repair of the VML injury (OST + VML + GRAFT) groups. (B) Quantified mineralized content from *μ* CT images at the fracture site. Values are mean ± SEM. Groups denoted with different letters are significantly different (*P* < 0.05), while those denoted with similar letters are statistically similar (*P* > 0.05).

**Figure 2:**
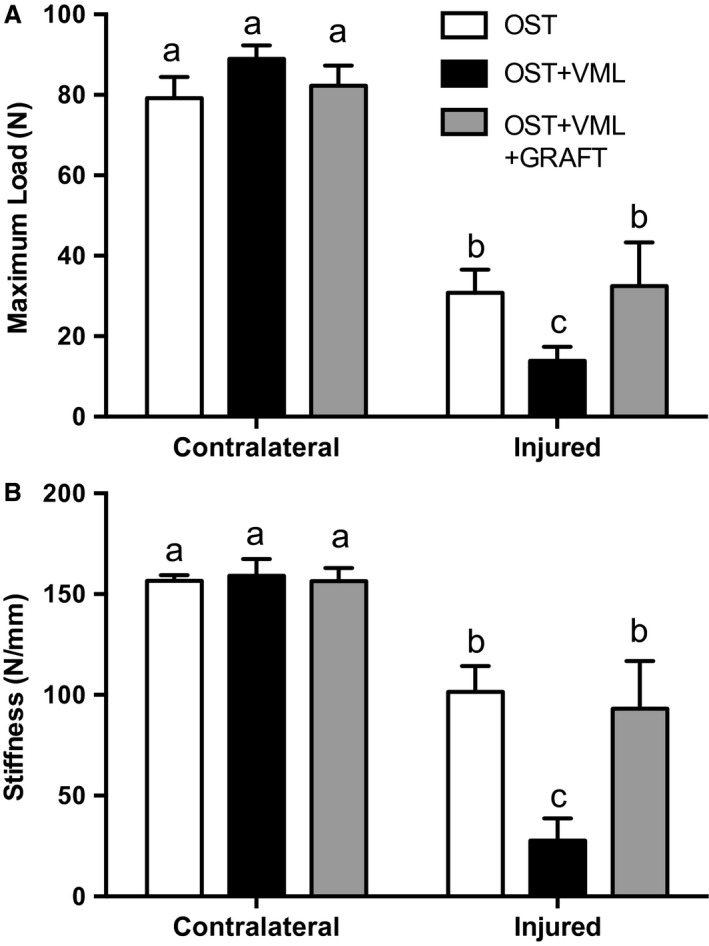
Tibia mechanical properties are augmented by repair of volumetric muscle loss (VML) injury with autologous minced muscle grafts (GRAFT). Three point bending was performed on tibia isolated 28 days postinjury and analyzed for (A) maximal load and (B) stiffness. Values are mean ± SEM. Groups denoted with different letters are significantly different (*P* < 0.05), while those denoted with similar letters are statistically similar (*P* > 0.05).

Tibia fracture healing was assessed at 28 days postinjury using *μ*CT and mechanical testing. *μ*CT analysis of the mineralized bone volume within the fracture callus indicated that VML impaired bone formation by 43% compared to OST. Graft repair of VML injury significantly improved bone formation compared to OST + VML to levels similar to OST‐only (Table 2; Fig. 1). Similar, observations were noted for mechanical characteristics of the injured tibia, wherein VML induced 55 and 73% reductions in maximal load and stiffness, respectively, and graft repair restored tibia maximal load and stiffness to OST‐only levels (Table 2; Fig. 2).

### TA muscle functional recovery

**Figure 3:**
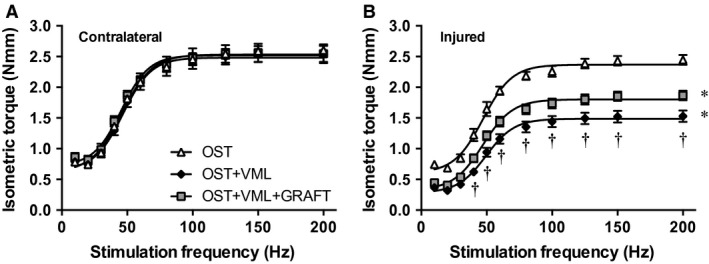
Tibialis anterior muscle isometric torque is partially restored after minced muscle graft repair. In vivo isometric torque was assessed as a function of stimulation frequency in (A) contralateral and (B) injured limbs 28 days postinjury. Values are mean ± SEM. * All values from each group are < respective values from OST; ^†^ values are < OST + VML + GRAFT;* P* < 0.05

**Figure 4:**
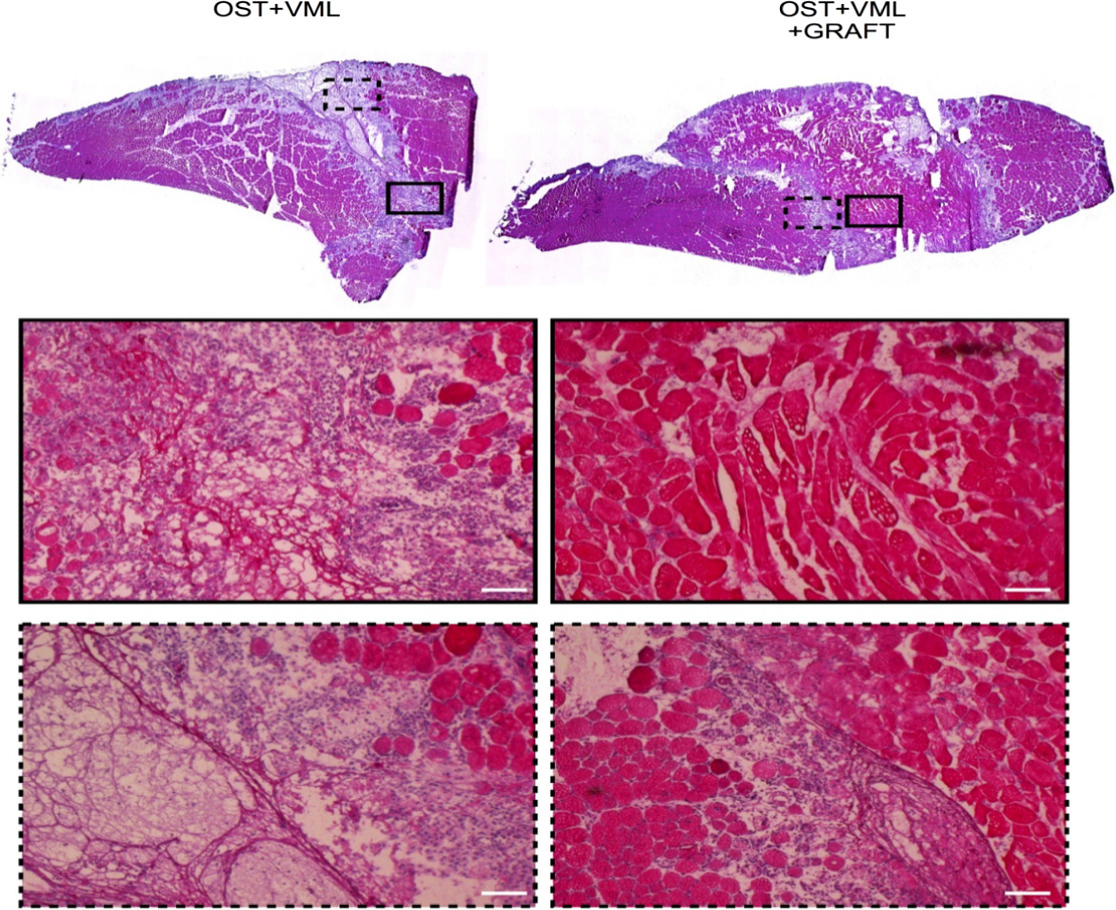
Autologous minced grafts promote muscle fiber regeneration. (A) Cross‐sections from the middle of the volumetric muscle loss (VML) defect region in tibialis anterior muscles harvested 28 days postinjury were stained with H&E and qualitatively analyzed. (B) Magnified regions specified by border outline (solid or dashed) from each respective muscle group. Scale bars = 100 *μ*m.

TA muscle isometric strength was measured in vivo as a function of stimulation frequency 28 days postinjury (Fig. 3). VML injury reduced the functional capacity of TA muscle compared to OST by 36–53% across all stimulation frequencies. Minced grafts improved muscle strength across a range of stimulation frequencies (40–200 Hz) compared to OST + VML, and presented a residual 23–41% functional deficit compared to OST (Table 2; Fig. 3). Muscle strength improvements imparted by minced graft repair were qualitatively attributed to the regeneration of muscle fibers (Fig. 4), as has been demonstrated in isolated VML injury models previously (Corona et al. 2013a; Garg et al. 2014b; Ward et al. 2015, 2016).

### TA muscle macrophage and T lymphocyte infiltration

**Figure 5:**
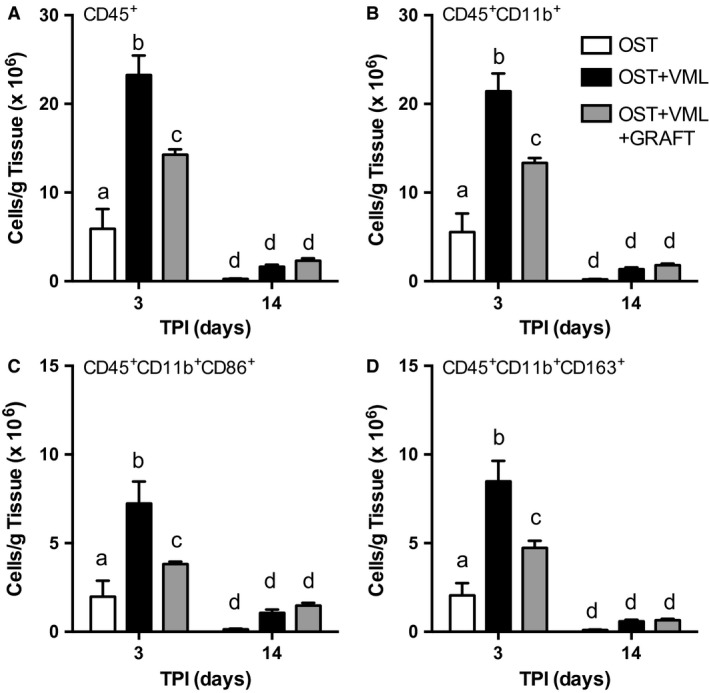
Acute myeloid cellular response to tibialis anterior (TA) muscle volumetric muscle loss (VML) injury is damped by muscle graft repair. Flow cytometry was performed in cell isolates from the middle third of the TA muscle 3 and 14 days postinjury (time postinjury; TPI). (A) hemaptopoietic cells (CD45^+^), (B) Monocytes/Macrophages (CD45^+^ CD11b^+^), (C) M1 macrophages (CD45^+^ CD11b^+^ CD86^+^), and (D) M2 macrophages (CD45^+^ CD11b^+^ CD163^+^) were quantified per group. Values are mean ± SEM. Groups denoted with different letters are significantly different (*P* < 0.05), while those denoted with similar letters are statistically similar (*P* > 0.05).

Previous observations have indicated that heightened muscle inflammation secondary to VML injury and disease associates with poor fracture healing (Abou‐Khalil et al. 2014; Hurtgen et al. 2016). Moreover, fracture healing after muscle flapping has been partly associated with attenuated fracture site inflammation (Brown et al. 2000). Therefore, we interrogated the immunomodulatory role of minced muscle grafts within VML injured muscle. Flow cytometry of mononucleated cells residing within the middle third of the TA muscle was performed at 3 and 14 days postinjury. At 3 days postinjury, VML injury resulted in a 293, 286, 263, and 312% increase in CD45^+^ hematopoietic lineage cells, CD45^+^CD11b^+^ monocytes (confirmed as >98% CD68^+^ macrophages), CD45^+^CD11b^+^CD86^+^ M1‐like macrophages, and CD45^+^CD11b^+^CD163^+^ M2‐like macrophages, respectively (Table 2; Fig. 5). Graft repair of the VML injury, significantly attenuated each of these cell populations leaving a residual elevation of 141, 141, 92, and 130%, respectively compared to OST only. At 14 days postinjury, each cell type was significantly reduced compared to 3 day values in each experimental group, and there was no effect of GRAFT treatment on cell populations at this time point.

**Figure 6:**
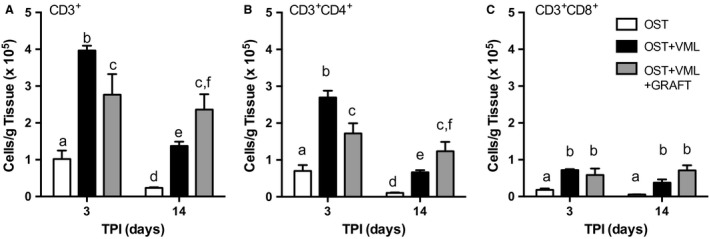
Autologous muscle graft repair differentially modulates T lymphocyte infiltration at acute and intermediate times postinjury. Flow cytometry was performed in cell isolates from the middle third of the tibialis anterior (TA) muscle 3 and 14 days after injury (time postinjury; TPI). (A) lymphocytes (CD3^+^), (B) T helper (CD3^+^ CD4^+^), (C) T cytotoxic (CD3^+^ CD8^+^) lymphocytes were quantified per group. Values are mean ± SEM. Groups denoted with different letters are significantly different (*P* < 0.05), while those denoted with similar letters are statistically similar (*P *> 0.05).

Flow cytometric analysis of T‐lymphocytes was also performed (Table 2; Fig. 6). The average ratio of CD3^+^ to CD45^+^CD11b^+^ cells over all groups was 0.02 and 0.12 at 3 and 14 days postinjury, respectively, indicating myeloid cells primarily drove the immune response. CD3^+^ T‐lymphocytes, CD3^+^CD4^+^ helper T cells, and CD3^+^CD8^+^ cytotoxic T cells were each elevated by 289, 283, and 296% at 3 days postinjury by VML injury, compared to OST. Graft repair attenuated the infiltration of CD3^+^ T lymphocytes (−30% compare to OST + VML), which was associated with a reduction of CD3^+^CD4^+^ helper T cells (−27%), but not CD3^+^CD8^+^ cytotoxic T cells. At 14 days postinjury, CD3^+^ T lymphocytes and CD3^+^CD4^+^ helper T cells both decreased from 3 days postinjury in OST and OST + VML groups, but not with graft repair (Fig. 6); and, at 14 days postinjury, these cell populations were elevated in OST + VML compared to OST (e.g., CD3^+^CD4^+^ cells were elevated by 635%), while OST + VML + GRAFT presented the highest presence of CD3^+^ T lymphocytes and CD3^+^CD4^+^ helper T cells of all groups (e.g., CD3^+^CD4^+^ cells were elevated by 1178% compared to OST). Regardless of time postinjury, CD3^+^CD8^+^ cytotoxic T cells were similarly elevated in OST+VML and OST + VML + GRAFT compared to OST only.

### TA muscle cytokine, chemokine, and growth factor protein expression

**Figure 7:**
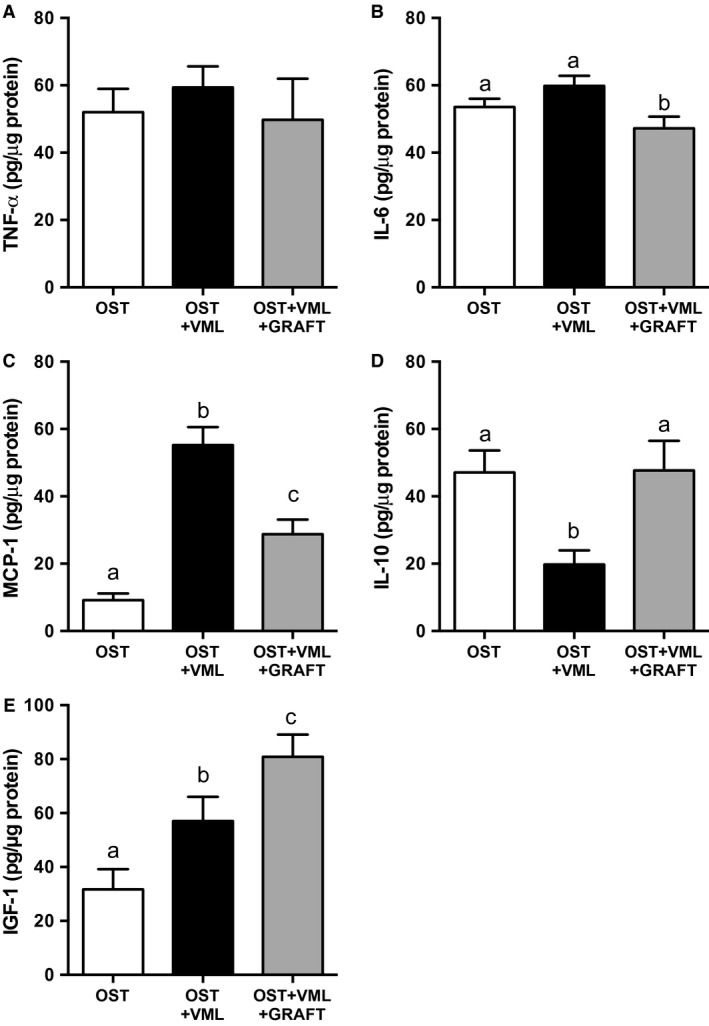
Tibialis anterior (TA) muscle inflammatory and growth factor concentrations a modulated by volumetric muscle loss (VML) injury and muscle graft repair. (A) TNF‐*α*, (B) IL‐6, (C) MCP‐1, (D) IL‐10, and (E) IGF‐1 were measured within the middle third of the TA muscle 3 days postinjury. Values are mean ± SEM. Groups denoted with different letters are significantly different (*P* < 0.05), while those denoted with similar letters are statistically similar (*P* > 0.05).

To further characterize the wound healing phenotype of the TA muscle, protein levels of select cytokines and chemokines were also assayed 3 days postinjury. While proinflammatory cytokines TNF‐*α* and IL‐6 were not significantly elevated in OST + VML groups compared to OST, the primary monocyte chemokine, MCP‐1 (CCL2), and the immune regulatory cytokine, IL‐10, were elevated (503%) and suppressed (58%), respectively, in OST + VML compared to OST (Fig. 7), creating a heightened proinflammatory environment. Graft repair of the VML injury partially attenuated MCP‐1 and completely restored IL‐10 to values detected in OST. Moreover, protein levels of an influential growth factor in musculoskeletal healing, IGF‐1, was elevated by 80% in OST + VML compared to OST only and further elevated with graft repair (42% greater than OST + VML; Fig. 7). Together, minced graft modulation of MCP‐1, IL‐10, and IGF‐1 within the muscle wound environment promoted a proregenerative environment.

### Circulating alarmins, leukocytes, and IGF‐1

**Figure 8:**
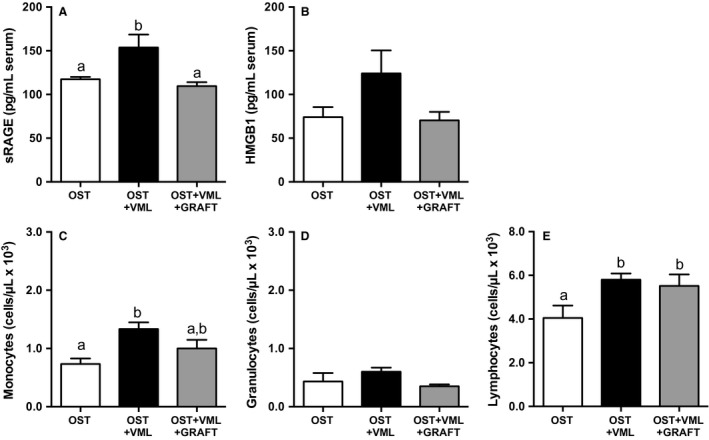
Heightened alarmin protein and circulating immune cells induced by concomitant volumetric muscle loss (VML) injury are selectively attenuated by muscle graft repair. Blood was collected 3 days postinjury and analyzed for (A and B) serum alarmin proteins or (C and D) circulating immune cells. Values are mean ± SEM. Groups denoted with different letters are significantly different (*P* < 0.05), while those denoted with similar letters are statistically similar (*P* > 0.05).

**Figure 9:**
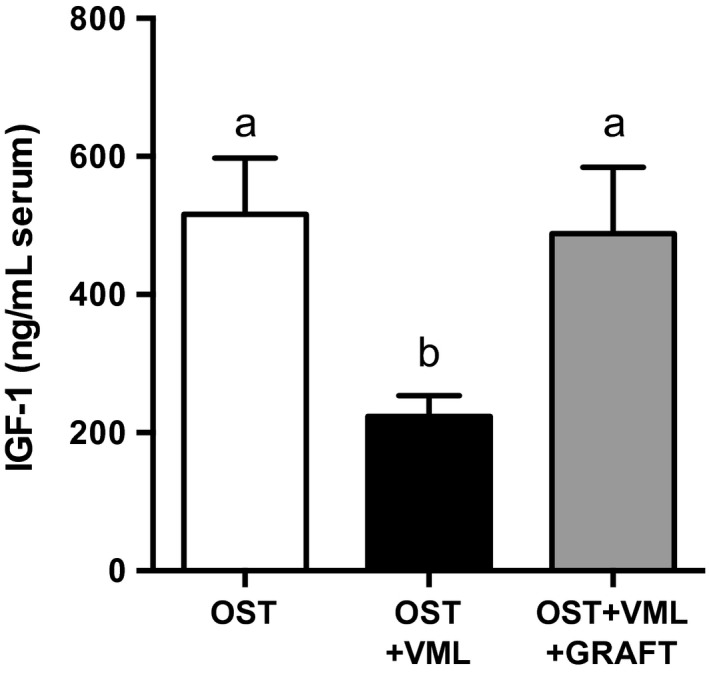
Autologous minced grafts augment circulating IGF‐1 concentrations. IGF‐1 was measured in blood serum collected 3 days postinjury. Values are mean ± SEM. Groups denoted with different letters are significantly different (*P* < 0.05), while those denoted with similar letters are statistically similar (*P* > 0.05).

Beyond the local traumatized skeletal muscle tissue, we interrogated if VML injury heightens systemic immune responses and damps systemic growth factor concentrations in a manner that may potentially deleteriously impact healing of other tissues (e.g., fractured bone); and, whether augmentation of traumatized musculature with minced grafts normalized these responses. As an initial step in provoking a systemic immune response to local tissue trauma, alarmins, such as soluble RAGE and HMGB1, are released from the traumatized tissue and initiates sterile immune responses through binding to TLR4 and RAGE receptor (Magna and Pisetsky 2014; Pilzweger and Holdenrieder 2015). OST + VML injury elevated circulating protein levels of soluble RAGE (31%) compared to OST, which returned to OST levels with graft repair of the VML injury (Fig. 8). In concert, a significant elevation of circulating monocytes (86%) in OST + VML compared to OST, was also normalized by graft repair (Fig. 8). Finally, serum IGF‐1 protein levels were reduced by 57% in OST + VML compared to OST, which graft repair of VML fully restored to OST levels (Fig. 9). In total, the nonrepaired and repaired muscle injury presented distinct local and systemic immune and growth factor profiles that each predictably associate with their different healing outcomes.

## Discussion

The negative impact of the loss of surrounding skeletal muscle on tibia fracture healing and chronic disability is well‐known (Utvag et al. 2002, 2003; Papakostidis et al. 2011; Willett et al. 2013; Corona et al. 2015; Hurtgen et al. 2016; Rivera and Corona 2016). To mitigate fracture healing complications, the most severe types of open fracture that involve a concomitant loss of overlying soft tissue are treated with fasciocutaneous or muscle flaps. However, these commonly used tissue flaps are not intended to restore the capacity of the traumatized muscle to produce force; and therefore despite limb salvage, dysfunction often persists (Garg et al. 2015). The most salient finding of this study is that a regenerative treatment of traumatized skeletal muscle (i.e., autologous minced muscle grafts) not only improves recovery of muscle strength as we previously demonstrated (Corona et al. 2013a; Garg et al. 2014b), but also tibia healing after osteotomy that would otherwise be impaired by concomitant VML injury. These findings highlight the clinical potential of acute direct skeletal muscle tissue therapies for comprehensive augmentation of “musculoskeletal” healing.

Evidence is emerging that exacerbated inflammation, secondary to severe soft tissue trauma is deleterious to concomitant fracture healing. Clinical evidence associates extremely heightened proinflammatory cytokine concentrations from soft tissue wound effluent with poor orthopaedic healing outcomes (Davis et al. 2011). Additionally, rodent polytrauma involving blunt chest trauma impairs femur fracture healing, which may be rescued by inhibition of immune Complement activation (Recknagel et al. 2011, 2012; Claes et al. 2012; Weckbach et al. 2012). Local exacerbated skeletal muscle inflammation has also been shown to impair nearby bone repair in dystrophic mice and after TA muscle VML injury (Abou‐Khalil et al. 2014; Hurtgen et al. 2016, 2017). Specifically, rodent TA VML injury has been shown to increase macrophage and T‐lymphocyte infiltration within the injured muscle and at the fracture site of the adjacent tibia and to delay recovery of tibia mechanical properties, which was ameliorated by systemic administration of the immunosuppressant drug FK506 (Hurtgen et al. 2016, 2017). Given that muscle injuries that present canonical immune and regenerative responses have been reported to not ultimately impair fracture healing (Kase et al. 1998; Utvag et al. 2003; Claes et al. 2006), it is highly plausible that the distinct VML injury‐induced immune response is a prominent mediator of musculoskeletal healing. For instance, VML injury presents a prolonged mixed M1/M2 rather than an acute M2‐converted macrophage response that is typically presented with other recoverable muscle injuries (e.g., crush injury) (McGeachie and Grounds 1987; Arnold et al. 2007; Nicholas et al. 2015). The anti‐inflammatory cytokine IL‐10, which aids in damping proinflammatory responses and promoting M2 activation (Katakura et al. 2004) was also suppressed in the nonrepaired VML group compared to osteotomy alone, suggesting that the shift to an anti‐inflammatory wound healing environment is delayed following VML injury. Additionally, VML injury induced elevation of DAMPs and in turn monocytes in the systemic circulation lend further support to VML injury protracting fracture site inflammation and disrupting fracture healing (Hurtgen et al. 2016, 2017).

Raising the importance of restoring inherent muscle‐bone interactions after severe musculoskeletal trauma, clinical evidence and animal models of open fracture indicate that muscle flaps are superior to their fasciocutaneous counterparts in supporting fracture healing (Harry et al. 2008, 2009; Chan et al. 2012). Skeletal muscle is known to provide multiple modes of support to bone regeneration (Hamrick 2011; Bonewald et al. 2013; Davis et al. 2015), and therefore it is possible that the viable myogenic tissue bed reconstituted by minced graft implantation may support fracture healing by various mechanisms. For instance, vascularization is undoubtedly crucial to successful diaphyseal fracture healing and myogenic precursor cells have been shown to migrate from surrounding skeletal to fracture bone, wherein they contribute in a primarily paracrine manner to support chondro‐ and osteo‐genesis (Liu et al. 2011; Abou‐Khalil et al. 2015). However, arguing against a vascular‐mediated mechanism of action, prior rodent open fracture studies involving TA VML injury have indicated that tibia vascularization is not a major limitation to bone regeneration (Utvag et al. 2002, 2003) and we have observed a similar up‐regulation of VEGF gene expression in fractured tibiae from OST‐only and OST + VML groups 3 days postinjury (B. T. Corona, unpubl. data). Additionally, re‐establishment of a highly vascularized fibrotic tissue bed following acellular biological scaffold implantation within a VML defect (Aurora et al. 2015) did not improve concomitant adjacent tibia bone formation (Pollot et al. 2016). Moreover, while minced grafts restore viable muscle progenitors within the VML defect (Ward et al. 2015; Corona et al. 2017), it is difficult to rationalize that a relatively small loss of the TA muscle (~20%) in the current study ablates such a large portion of the satellite cell pool that cannot be compensated for by the bulk of the surrounding musculature (e.g., triceps surae muscles) that is left intact. Therefore, specific characteristics of this open fracture model points toward immune and autocrine/paracrine responses in nonrepaired and minced graft‐repaired VML injured muscles as potential primary mediators of fracture healing.

It is interesting to speculate that musculoskeletal healing outcomes dictated by the repair status of the VML injury observed in this study is related to immunophysiological interactions involving inflammatory cytokine and IGF‐1 signaling (Kelley 2004). The proinflammatory cytokine IL‐1ß has been shown to induce resistance to IGF‐1 mediated myogenesis in cultured myoblasts, in a manner dependent on IL‐10 activity (Strle et al. 2008). In turn, IGF‐1 has been shown to reduce TLR‐4 expression and NF*κ*B associated proinflammatory cytokine production (i.e., TNF‐*α* and IL‐6) in cultured myotubes (Lee 2011). Together, these findings highlight the balanced nature of host defense and regeneration. In this study, we demonstrate robust skeletal muscle regeneration with minced graft repair that is accompanied by attenuated acute macrophage and T‐lymphocyte infiltration, as well as elevated IL‐10 and IGF‐1 compared to nonrepaired VML injured muscle. Thus, it is likely that the combination of minced grafts modulating the inflammatory and hormonal milieu and delivering basal lamina and muscle progenitors created an environment permissive to skeletal muscle regeneration within the VML defect. What is more, we observed that minced graft repair effectively normalized the systemic IGF‐1 response that was suppressed by nonrepaired VML injury, putatively indicating the restoration skeletal muscle paracrine support of fracture healing (Meinel et al. 2003; Fowlkes et al. 2006; Hamrick et al. 2010; Tonkin et al. 2015).

We previously observed a prolonged presence of CD4^+^ and CD8^+^ T cells within VML injured muscle as time progressed (Hurtgen et al. 2016). We initially hypothesized that the delayed recession of T cells from the injury site was contributing to inflammatory pathology. Contradictory to these suppositions, here we observed a sustained number of CD4^+^ and CD8^+^ T cells within the muscle defect in graft‐repaired muscle tissue. The specific phenotype of the T cells at these various time points could explain this unexpected but beneficial result. For example, while Th1 and Th17 cells generally induce proinflammatory immune responses, Th2 cells are anti‐inflammatory and contribute to wound healing as a normal part of tissue repair (Allen and Wynn 2011). In addition, regulatory T cells (Tregs) also CD4^+^, are known to actively suppress proinflammatory responses (Schiaffino et al. 2017), and are linked to regeneration of skeletal muscle by acting on satellite cells (Burzyn et al. 2013; Matta et al. 2014; Arpaia et al. 2015). CD8^+^ T cells also have a positive impact on satellite cell proliferation (Zhang et al. 2014). Future studies are needed to define the T‐lymphocyte populations in non‐ and minced graft‐repaired muscle and to then determine their roles in musculoskeletal healing.

Clinical application of autologous minced grafts without adjunctive support (e.g., a myoconductive expansion material) is clearly limited to the repair of small defects. Thus, the current collective findings do not necessarily ascribe a specific therapy for clinical use, but rather conservatively indicate the intrinsic value of promoting a regenerative environment within traumatized skeletal muscle tissue for the benefit of fracture healing and functional recovery. While we did not previously observe a benefit to musculoskeletal healing with acellular biological scaffold repair of VML injury (Pollot et al. 2016), it is possible that other developing therapies for VML injuries (Rossi et al. 2011; Corona et al. 2012; Van Dusen et al. 2014; Grasman et al. 2015) may provide the necessary regenerative cues to ameliorate challenged bone and muscle regeneration.

Deterioration of skeletal health is associated with various progressive muscle‐related conditions and diseases, such as sarcopenia, polymyositis, and Duchenne Muscular Dystrophy (Bonewald et al. 2013; Abou‐Khalil et al. 2014; Maruotti et al. 2014). Similarly, polytrauma and severe musculoskeletal trauma also present organ damage and muscle‐associated perturbation of fracture healing (Papakostidis et al. 2011; Recknagel et al. 2011, 2012; Hurtgen et al. 2016). In considering directional muscle to bone communication, vascular, cellular, paracrine, and mechanical modes of support are all involved in the maintenance, plasticity, and regeneration of local skeletal tissue (Davis et al. 2015), and predictably progressive deterioration or abrupt cessation of these modes of communication contributes to the development of musculoskeletal pathobiology. The overwhelming directional response of the skeletal muscle milieu and coinciding musculoskeletal healing responses (Table 2) signify the role of acute exacerbated skeletal muscle inflammation in contributing and potentially coordinating complicated fracture healing in the absence of infection. Tempered to the rodent model interrogated, these findings support the continued development of acute regenerative skeletal muscle therapies to improve musculoskeletal healing outcomes following severe open fracture.

## Conflict of Interest

The opinions or assertions contained herein are the private views of the authors and are not to be construed as official or as reflecting the views of the Department of the Army or the Department of Defense. The authors have no perceived or potential conflicts of interest to disclose, financial or otherwise.
